# The Symbiotic Biofilm of *Sinorhizobium fredii* SMH12, Necessary for Successful Colonization and Symbiosis of *Glycine max* cv Osumi, Is Regulated by Quorum Sensing Systems and Inducing Flavonoids via NodD1

**DOI:** 10.1371/journal.pone.0105901

**Published:** 2014-08-28

**Authors:** Francisco Pérez-Montaño, Irene Jiménez-Guerrero, Pablo Del Cerro, Irene Baena-Ropero, Francisco Javier López-Baena, Francisco Javier Ollero, Ramón Bellogín, Javier Lloret, Rosario Espuny

**Affiliations:** 1 Departamento de Microbiología, Facultad de Biología, Universidad de Sevilla, Sevilla, Spain; 2 Departamento de Biología, Facultad de Ciencias, Universidad Autónoma de Madrid, Madrid, Spain; East Carolina University School of Medicine, United States of America

## Abstract

Bacterial surface components, especially exopolysaccharides, in combination with bacterial Quorum Sensing signals are crucial for the formation of biofilms in most species studied so far. Biofilm formation allows soil bacteria to colonize their surrounding habitat and survive common environmental stresses such as desiccation and nutrient limitation. This mode of life is often essential for survival in bacteria of the genera *Mesorhizobium*, *Sinorhizobium*, *Bradyrhizobium*, and *Rhizobium.* The role of biofilm formation in symbiosis has been investigated in detail for *Sinorhizobium meliloti* and *Bradyrhizobium japonicum*. However, for *S. fredii* this process has not been studied. In this work we have demonstrated that biofilm formation is crucial for an optimal root colonization and symbiosis between *S. fredii* SMH12 and *Glycine max* cv Osumi. In this bacterium, *nod*-gene inducing flavonoids and the NodD1 protein are required for the transition of the biofilm structure from monolayer to microcolony. Quorum Sensing systems are also required for the full development of both types of biofilms. In fact, both the *nodD1* mutant and the lactonase strain (the lactonase enzyme prevents AHL accumulation) are defective in soybean root colonization. The impairment of the lactonase strain in its colonization ability leads to a decrease in the symbiotic parameters. Interestingly, NodD1 together with flavonoids activates certain quorum sensing systems implicit in the development of the symbiotic biofilm. Thus, *S. fredii* SMH12 by means of a unique key molecule, the flavonoid, efficiently forms biofilm, colonizes the legume roots and activates the synthesis of Nod factors, required for successfully symbiosis.

## Introduction

Rhizobia are bacteria that interact with the roots of leguminous plants to develop symbiotic nodules when environmental nitrogen is limited. In these symbiotic structures atmospheric nitrogen is reduced to ammonium making it available to the plant. This process requires the exchange of molecular signals between both members of the symbiosis. Legume roots exude flavonoids which induce, via a mechanism involving the NodD family of transcription activators, the biosynthesis and secretion of strain-specific lipo-chito-oligosaccharides, also known as nodulation factors (Nod factors), which trigger the initiation of nodule organogenesis [1]. Plant flavonoids, besides inducing Nod factor production, attract the bacteria to the legume root [2]; induce type III secretion machinery via NodD1 by activation of the transcriptional regulator TtsI [3], [4]; and activate the rhizobial Quorum Sensing (QS) systems [5].

QS is defined as a regulation mode of bacterial gene expression in response to changes in their population density, which is mediated by small diffusible signal molecules called autoinducers (AI) [6], [7]. QS-regulated genes are involved in adaptive changes in the physiology of the bacterial population, which modify their behaviour when a certain cell density is reached. QS modulates a broad variety of phenotypes, such as biofilm formation, toxin and exopolysaccharide production, virulence, plasmid transfer and motility, which usually are essential for successful establishment of a symbiotic or a pathogenic relationship with the eukaryotic hosts [8], [9], [10], [11], [12]. In plant-associated bacteria QS coordinates the expression of genes involved in virulence, colonization and symbiosis [13].

In all species studied so far, bacterial surface components (flagella, lipopolysaccharides and especially exopolysaccharides) in combination with the presence of bacterial QS signals are crucial for the formation of biofilms [12]. Biofilms are defined as bacterial communities surrounded by a self-produced polymeric matrix, and attached to an inert or a biotic surface [14]. In the course of biofilm formation, the initial reversible attachment to the surface is followed by an irreversible attachment and multiplication of the bacteria forming microcolonies that develop mature communities with a three-dimensional structure, in some cases permeated by channels, which act as the biofilm circulatory system. All these processes are coordinated by bacterial QS systems [14], [15]. Many rhizobia have been described as forming microcolonies or biofilms when they colonize legume roots. These biofilms are mainly composed of water and bacterial cells. However, the three-dimensional structure of the biofilm is due to an extracellular matrix, which is formed by exopolysaccharides (EPS) [16]. In *S. meliloti*, the biofilm formation is affected and/or regulated by nutritional and environmental conditions [17], [18]; exopolysaccharides and flagella [19]; ExoR with the ExoS–ChvI two-component system [20]; Nod factors synthesized by *nod* genes [19]; and regulation of exopolysaccharide biosynthesis by means of QS systems [21]. Development of biofilms by rhizobia is crucial to overcoming environmental stresses and in certain species the biofilm formation is clearly an important feature of symbiotic ability [12], [19], [22], [23], [24]. *S. fredii* SMH12, a wide host-range rhizobium that nodulates soybean and dozens of other legumes, produces at least three AHLs, *N*-octanoyl homoserine lactone (C8-HSL), 3-oxo *N*-octanoyl homoserine lactone (3-oxo-C8-HSL) and *N*-tetradecanoyl homoserine lactone (C14-HSL). In the presence of the flavonoid genistein, an activator of its nodulation genes, the overall production of AHLs is enhanced, being detected the C14-HSL only in bacterial cultures supplemented with inducing flavonoids [5]. SMH12 possesses at least one gene, *traI*, which is responsiblefor the synthesis of the 3-oxo-C8-HSL [5]; and at least two non-identified *luxI*-type gene involved in the synthesis of the C8-HSL and the C14-HSL. Furthermore, in this bacterium the AHLs and the QS systems are involved in the biofilm formation on the abiotic surface [25].

In this work, the role and regulation of biofilm formation in *S. fredii* SMH12 have been studied during symbiosis with *Glycine max* cv. Osumi. For this purpose, the wild-type strain, a *nodD1* mutant and a lactonase overproducing strain of SMH12 were constructed. The lactonase enzyme hydrolyzes the ester bond of the homoserine lactone ring of acylated homoserine lactones preventing these signalling molecules from binding to their target transcriptional regulators. Both *nodD1* and lactonase strains showed an impaired ability for colonization of *Glycine max* roots with respect to the wild-strain, probably due to an abnormal symbiotic biofilm formation which determines a less effective symbiosis. Interestingly, QS-biofilm formation and effective nodulation are connected through flavonoids, since these molecules initiate the molecular dialogue between bacteria and plants and allow the symbiotic biofilm formation. In summary, this report unequivocally demonstrates that the development of biofilm is crucial for successful root colonization and optimal symbiosis in *S. fredii* SMH12.

## Materials and Methods

### Strains and media

Bacterial strains and plasmids used in this work are listed in Table 1. *S. fredii* SMH12 and derivative strains were grown at 28°C in tryptone-yeast extract (TY) medium [26], yeast extract mannitol (YM) medium [27] with a lower mannitol concentration (3 g l^−1^) and low-phosphate minimal glutamate mannitol (MGM) medium [21], supplemented when necessary with genistein 3.7 µM as inducing flavonoid, umbelliferone 6.2 µM as non-inducing flavonoid, or with commercial AHLs [25]. *Agrobacterium tumefaciens* NT1 (pZLR4) was grown at 28°C in YM with carbenicillin (100 µg ml^−1^) and gentamicin (30 µg ml^−1^). *Escherichia coli* strains were cultured in Luria-Bertani (LB) medium (28) at 37°C. When required, the media were supplemented with the appropriate antibiotics as described by Lamrabet et al. [29]. Commercial AHLs were dissolved in methanol and used at different concentrations. Flavonoids and AHLs were purchased from Fluka (Sigma-Aldrich, USA).

**Table 1:** Resistance phenotypes: Gm^R^, Km^R^, Nx^R^, Ap^R^ and Tc^R^, gentamicin, kanamycin, nalidixic acid, ampicillin and tetracycline, respectively.

Strain or plasmid	Relevant properties	Reference
*Agrobacterium tumefaciens*NT1 (pZRL4)	*A.tumefaciens* without pTiC58;with pZRL4, which carries thefusion *traG::lacZ* and the gene *traR*, Gm^R^	[13]
*Escherichia coli*DH5α	*SupE44*, Δ*lacU169*, 5hsdR17,*recA1*, *endA1*, *gyrA96*, *thi-1*, *relA1*, Nx^R^	[25]
*Sinorhizobium* *fredii* SMH12	Wild-type strain, Ap^R^	[40]
SMH12 (pME6000)	SMH12 with the plasmid pME6000, Tc^R^	This work
SMH12 (pME6863)	SMH12 with the plasmid pME6863, whichcarries the lactonase gene, Tc^R^	This work
SMH12 (pMP2463)	SMH12 with the plasmid pMP2463, whichcarries the gene of the GFP, Gm^R^	This work
SMH12 (pMP6000)(pMP2463)	SMH12 with the plasmids pME6000 and pMP2463,which carry the empty plasmid and thegreen fluorescent protein, respectively, Tc^R^ Gm^R^	This work
SMH12 (pMP6863)(pMP2463)	SMH12 with the plasmids pME6863 and pMP2463,which carry the gene that encode the lactonaseand the green fluorescent protein, respectively, Tc^R^ Gm^R^	This work
SVQ648	SMH12 *nodD1::lacZ*-Gm^R^	This work
SVQ648 (pMP2463)	SVQ648 with the plasmid pMP2463, whichcarries the gene of the GFP, Gm^R^	This work
pBBR1MCS-5	Broad-host-range cloning vector, Gm^R^	[41]
pK18mobsacB	Cloning vector (suicide in rhizobia), Km^R^	[42]
pME6000	Broad-host-range cloning vector, Tc^R^	[43]
pME6863	pME6000*::aiiA*, plasmid carrying thelactonase gene, Tc^R^	[44]
pMP2463	pBBR-MCS-5*::egfp-1,* plasmid carryingthe green fluorescent protein gene, Gm^R^	[45]
pMUS534	Plasmid pK18mob derivative containing*nodD1::lacZ*-Gm^R^	[46]
pRK2013	Helper plasmid, Km^R^	[47]

Plasmids were transferred from *E. coli* to SMH12 by conjugation as described by Simon [30] using plasmid pRK2013 as helper. Recombinant DNA techniques were performed according to the general protocols of Sambrook et al. [28]. For hybridization, DNA was blotted to Hybond-N nylon membranes (Amersham, UK), and the DigDNA method of Roche was employed according to the manufacturer’s instructions. PCR amplifications were performed as previously described [31]. Using this methodology the plasmid pMUS534 was employed for the homogenotization of the mutated version of the *nodD1* gene in *S. fredii* SMH12, generating the mutant strain SVQ648. The double recombination event was confirmed by Southern blotting (data not shown).

For the growth curves of the different strains, bacteria were grown in 5 ml of YM medium to early stationary phase and then diluted to OD_600_ values around 0.03 in fresh YM medium with or without flavonoids. Growth was monitored by measuring the OD_600_ for at least 46 h. Each experiment was performed two days, eight replicates each day.

### RNA isolation, cDNA synthesis and Quantitative RT-PCR

Bacterial RNA was extracted from bacterial cultures from the microtiter plates in the same conditions employed for the biofilm assays and described below. For the RNA extraction the PowerBiofilm RNA Isolation Kit was employed following the manufacturer’s instructions (MO BIO, USA). Three independent RNA extractions were performed.

To quantify the expression of the *S. fredii* SMH12 *traI* gene using quantitative RT-PCR, primers rt-traI-F and rt-traI-R described by Pérez-Montaño et al. [5] were used. Expression was calculated relative to bacteria grown without flavonoid. The *S. fredii* SMH12 RNA *16S* gene was used as internal control to normalize gene expression. RNA *16S* primers used are described in the same work [5]. The expression data shown are the mean (± standard deviation of the mean) for three biological replicates. The fold change in the target gene, normalized to RNA *16S*, and relative to gene expression in the culture without flavonoids was calculated. PCR was conducted on the LightCycler 480 (Roche, Switzerland) with the following conditions: 95°C, 10 min; 95°C, 30 s; 50°C, 30 s; 72°C, 20 s; forty cycles, followed by the melting curve profile from 60 to 95°C to verify the specificity of the reaction. The threshold cycles (Ct) were determined with the iCycler software and the individual values for each sample were generated by averaging three technical replicates that varied less than 0.5 per cycle.

### Confocal laser scanning microscopy

The different events of biofilm formation were visualized by confocal laser scanning microscopy using a method described by Russo et al. [32]. Bacterial cultures were placed in 8 well chambered cover glass slides containing a borosilicate glass base 1 µm thick (Thermo Fischer Scientific Inc., USA) for 4 days without shaking. To avoid desiccation, the chambers were incubated in a humid sterilized petri dish. Confocal microscope image capture was carried out with Leica TCS SP2 (Leica Microsystems, Germany). *In silico* 3D reconstruction analysis was executed using the computer program ImageJ (Java, USA).

### Biofilm formation assay

The biofilm formation assay was based on the method described by O′Toole and Kolter [33] with modifications [34]. Cultures were grown in 5 ml of low-phosphate MGM medium, diluted to an OD_600_ of 0.2, with or without flavonoids and AHLs, and inoculated with 100 µl aliquots and placed on polystyrene microtiter plates, U form (Deltalab S.L., Spain). The plates were inverted and incubated at 28°C for 7 days with gentle rocking. Cell growth was analyzed by measuring OD_600_ using a microtiter reader Synergy HT (Biotek, USA). The culture in each well was removed carefully; the wells were dried, washed three times with 0.9% NaCl and dried again. Biofilms in each well were stained with 100 µl of 0.1% crystal violet for 20 minutes, then washed with water three times and dried again. Finally, 100 µl of 96% ethanol were added to each well and the OD_570_ was measured. Every experiment was performed six times with eight replicates each time.

### Quantification of overall AHLs

Supernatants of the rhizobial strains grown for 7 days in 96 wells on U shaped polystyrene microtiter plates (Deltalab S.L., Spain) were collected and sterilized by microfiltration. To determine the overall autoinducer production in each condition the method described by Pérez-Montaño et al. was used. [5]. Briefly, 25 µl, 2.5 µl or 0.25 µl of the supernatants were mixed with YM to obtain a final volume of 2.5 ml reaching supernatant concentrations of 1%, 0.1% and 0.01% (v/v). The mixtures were inoculated with approximately 10^7^ cells ml^−1^ of the *A. tumefaciens* NT1 (pZLR4), incubated with shaking for 12 h at 28°C and assayed for β-galactosidase activity [35]. As controls, 125 µl of distilled water or 125 µl of *N*-(3-oxo-hexanoyl)-l-homoserine lactone (3-oxo-C6-HSL) 5.5 µM, were used. To obtain the standard curve with synthetic AHLs, 125 µl of 3-oxo-C8-HSL, C8-HSL or C14-HSL at different concentration were added. The experiments were repeated independently five times with 3 replicates.

### Thin Layer Chromatography

For TLC analysis, cultures previously removed from each well of the microtiter plate were extracted with the same volume of dichloromethane, evaporated to dryness and analyzed by thin-layer chromatography as described by Pérez-Montaño et al. [25]. Briefly, 1 µl of each culture extract was loaded on TLC plates (HPTLC plates RP-18 _F254s_ 1.13724 and 1.05559, Merck, Germany) using methanol:water (60∶40 v/v) as eluent, dried and overlaid with a soft agar culture of the biosensor *A. tumefaciens* NT1 (pZLR4).

### Plant assays

Nodulation assays on *Glycine max* (L.) Merrill cultivar Osumi were performed as described by de Lyra et al. [36]. Plants were inoculated with approximately 5×10^8^ bacteria and were grown in Leonard jars with Fåhraeus nutrient solution [27] for 42 days with a 16 hour-photoperiod at 26°C in light and 18°C in the dark with 70% of humidity. Shoots were dried at 70°C for 48 h and weighed. Experiments were performed three times. For root colonization assays and microscopy, seeds were surface sterilized, germinated and inoculated with a modified method described in Pérez-Montaño et al. [25]. Briefly, *Glycine max* (L.) Merrill cultivar Osumi seeds were soaked for 30 seconds in 96% ethanol and for 8 minutes in commercial bleach. Then, seeds were washed repeatedly with sterilized distilled water, germinated and checked for sterility in LB medium. Each plant was inoculated with approximately 5×10^8^ bacteria. Plants were grown under hydroponic controlled conditions in Fåhraeus solution for 7 days in the same conditions as those mentioned above.

### Epifluorescence and scanning electron microscopy

Epifluorescence microscopy assays were carried out with *S. fredii* SMH12 and derivatives carrying GFP expressing plasmid pME2463. After growth, roots were excised (one centimetre of the proximal area of the main root and the last centimetre of a lateral root) and thoroughly washed with water to eliminate any loosely attached bacteria. Afterwards, roots were soaked for 3 minutes in 0.5% crystal violet to avoid auto-fluorescence, following 3 washes with distilled water. Microscopy analysis was carried out using an Olympus BH2-FRCA microscope with 40x and 100x magnifications (Olympus, Japan). Images of GFP-labelled bacterial cells were obtained by using a filter set consisting of a 400 to 490 nm (BP490) bandpass exciter, a 505 nm dicroic filter and a 530 nm longpass emitter (EO530). In all cases, exposure length of red and green channels was 30 seconds.

For electron scanning microscopy, 7 day-soybean plants were collected and the roots were excised as described above. Sample preparation and visualization were done according to Barahona *et al.* [37].

### Root attachment

Whole plants were carefully taken from the hydroponic solution and roots were excised and thoroughly washed with water to eliminate any loosely attached bacteria. Remaining attached bacteria were recovered from the root by vortexing (one centimetre of the proximal area of the main root or the last centimetre of a lateral root) for 2 min in a tube containing YM medium and plating the appropriate dilutions on YM medium plates. Experiments were performed three times with three plants per assay.

## Results

### Nodulation Test

In a previous work [5] we described that certain plant flavonoids, besides inducing synthesis of Nod factors, increase the overall production of QS signals in *S. fredii* SMH12. In many bacteria, these systems regulate a broad variety of phenotypes which are important for the successful establishment of a relationship with the eukaryotic hosts. For this reason, a nodulation test was performed to elucidate if SMH12 QS systems are regulating any important phenotype during the symbiosis with *Glycine max*. The symbiotic properties of the wild-type strain, SVQ648 (SMH12 *nodD1*::LacZ::Gm^R^), hereafter *nodD1* mutant, SMH12 (pME6863), hereafter lactonase strain (this enzyme prevents AHL accumulation), and SMH12 (pME6000), which carries the empty plasmid, were determined in plant infection tests with soybean cultivar Osumi which are effectively nodulated by the wild-type strain *S. fredii* SMH12.

As expected, the *nodD1* mutant did not induce nodule development due to its inability to produce Nod factors, which are responsible for the formation of these structures in the soybean roots. Consequently, the plant top dry mass was significantly lower (a 60%, where * is the statistical significance of the differences observed using the Mann–Whitney non-parametric test) in plants inoculated with the *nodD1* mutant than in those inoculated with the parental strain SMH12. In the cases of the plants inoculated with the lactonase strain, the top dry mass, number of nodules and fresh mass of nodules formed were lower than in the wild-type strain (20%, 40% and 35% respectively). Only in the case of the number of nodules were these differences statistically significant (where * is the statistical significance of the differences observed using the Mann–Whitney non-parametric test) (Table 2). To confirm that differences in this strain are not due to the presence of the plasmid, the symbiotic parameters of SMH12 carrying the empty plasmid (pME6000) were studied. No changes were observed with respect to the wild-type strain. In summary, these results indicate that QS systems could regulate some important phenotypes during the symbiosis with *Glycine max*.

**Table 2:** Plant responses to inoculation of *Glycine max* cv. Osumi with *S. fredii* SMH12 and derivatives.

Inoculant	Number of nodules	Fresh mass of nodules (g)	Plant-top dry mass (g)
None	0±0*	0±0*	0.48±0.11*
SMH12	124.30±12.06	1.56±0.20	1.34±0.18
SVQ648	0±0*	0±0*	0.46±0.10*
SMH12 (pME6863)	76.20±16.02*	1.04±0.29	1.10±0.30
SMH12 (pME6000)	131.00±22.68	1.60±0.38	1.35±0.25

Data represent means ± sd of six soybean jars. Each jar contained two soybean plants. Determinations were made six weeks after inoculation. Mutant *nodD1* and the lactonase strain parameters were individually compared with the parental strain SMH12 parameters by using the Mann-Whitney non-parametric test. Values tagged by * are significantly different at the level α = 5%.

### Plant root colonization

As commented in the introduction, in plant-associated bacteria one of the processes controlled by bacterial QS systems is the root colonization. For this reason three independent assays to study root colonization were carried out: root attachment quantification, observation studies of root by epifluorescence microscopy and electronic barrier microscopy. In these studies, proximal and lateral roots were analyzed separately to test differences in bacterial colonization in the whole root.

Root attachment assays showed a statistically significant reduction in the bacterial number per cm on the proximal area of the main root in the lactonase strain, the detected bacteria being around 10-fold less than in the other two strains. In lateral roots, colonization differences (statistically significant) were even more pronounced, these differences being 100-fold less than in the wild-type strain. A slight reduction in the number of bacteria per cm on the lateral root was also observed in the *nodD1* mutant (10-fold less than SMH12). No changes in root attachment were observed in plants inoculated with the SMH12 strain carrying the empty plasmid with respect to those inoculated with the wild-type strain (Table 3). No differences in the bacterial growth-curves were detected among the four strains (data not shown).

**Table 3:** *Glycine max* cv. Osumi root attachment.

Inoculant	CFU/cm of proximal root	CFU/cm of lateral root
None	0±0*	0±0*
SMH12	1.68±0.18×10^5^	1.22±0.29×10^4^
SVQ648	1.10±0.42×10^5^	3.83±0.5×10^3^*
SMH12 (pME6863)	5.00±0.10×10^4^*	8.45±0.46×10^2^*
SMH12 (pME6000)	1.55±0.27×10^5^	1.20±0.39×10^4^

Soybean plants were inoculated with the test strains, one centimetre of the proximal root and of the lateral roots of each plant were collected after 7 days, and the bacteria present were resuspended and plated. Data are the mean ± SD of at least three independent experiments performed in triplicate. Mutant *nodD1* and the lactonase strain attachment were individually compared with the parental strain SMH12 attachment by using the Mann-Whitney non-parametric test. Values tagged by * are significantly different at the level α = 5%.

**Figure 1:**
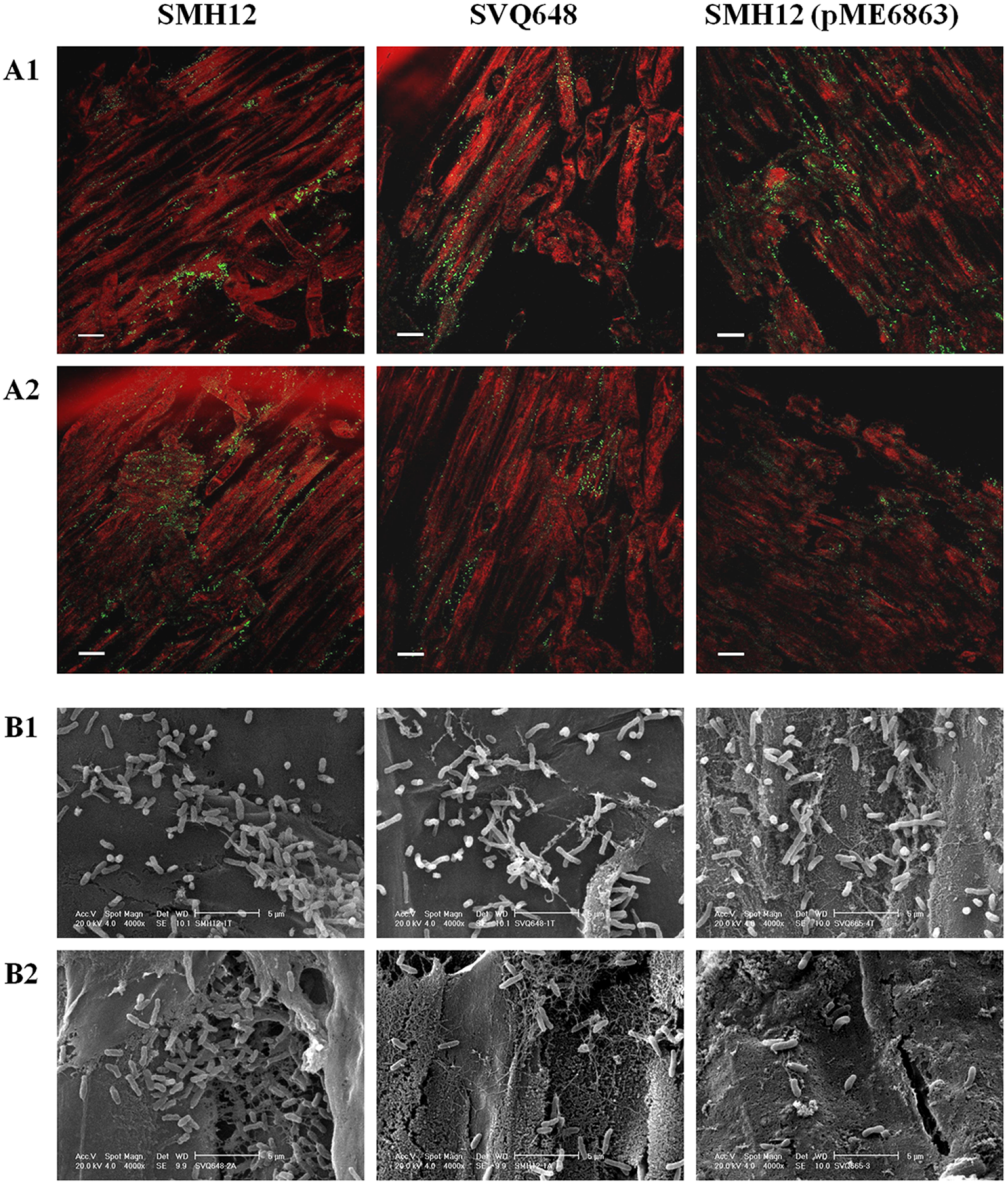
Visualization of the soybean root colonization. A. Epifluorescence microscopy analysis of the colonization of the soybean rhizosphere by gfp-tagged bacteria [SMH12, SVQ648, SMH12 (pME6863)]. Roots were visualized 7 days after inoculation. 1. Proximal root. 2. Lateral roots. Bar, 100 µm. B. Scanning microscopy analysis of the colonization of the soybean rhizosphere by SMH12, SVQ648 and SMH12 (pME6863). Roots were visualized 7 days after inoculation. 1. Proximal root. 2. Lateral roots. Bar, 5 µm. SMH12: wild-type, SVQ648: *nodD1* mutant, SMH12 (pME6863): lactonase strain.

Epifluorescence microscopy and electronic barrier microscopy assays (Fig. 1) showed that in lateral roots of plants inoculated with *nodD1* mutant and lactonase strain, the soybean root surface presented lower bacterial density with respect to the wild-type strain. Interestingly, the wild-type strain seems to be also distributed more clustered than the other two strains along the main root and especially in the lateral roots, which could indicate different biofilm structures in these strains. All these results suggest that the *nodD1* mutant and especially the lactonase strain are defective in root colonization.

### Influence of inducing flavonoids on biofilm structure

**Figure 2:**
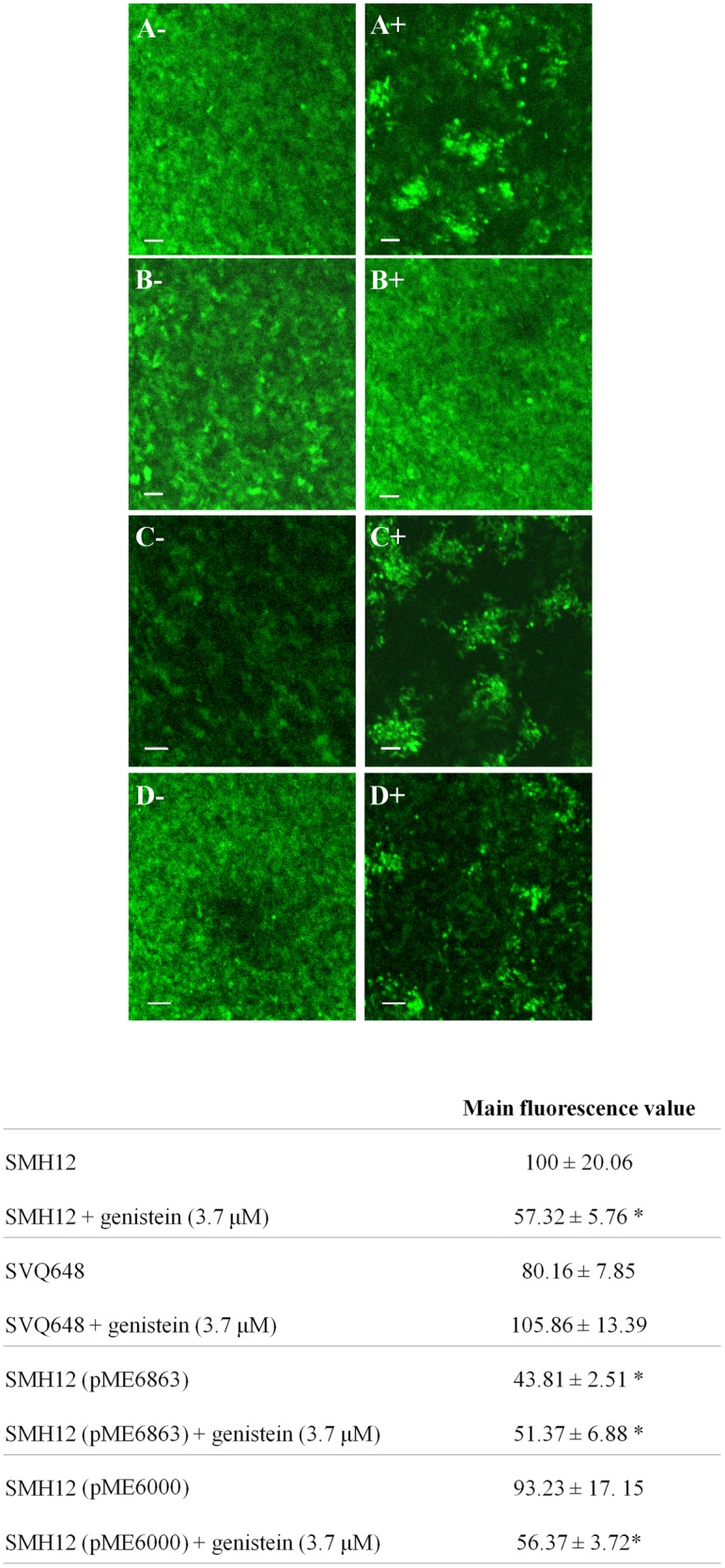
Biofilm structure of *S. fredii* SMH12 and derivatives on glass surfaces: reconstruction of the Z-stacks and measure of the surface coverage. Main fluorescence value of the wild-type strain was arbitrarily given a value of 100. Averages and standard deviations of five randomized optical fields per strain corresponding to two independent experiments are shown. The asterisks indicate a significant different at the level α = 5% with respect to wild-type strain by using the Mann-Whitney non-parametrical test. Left side corresponds to cultures without flavonoids. Right side corresponds to cultures with inducing flavonoid. A. SMH12. B. SVQ648. C. SMH12 (pME6863). D. SMH12 (pME6000). Bar, 20 µm. SMH12: wild-type, SVQ648: *nodD1* mutant, SMH12 (pME6863): lactonase strain. SMH12 (pME6000): carrying the empty plasmid.

**Figure 3:**
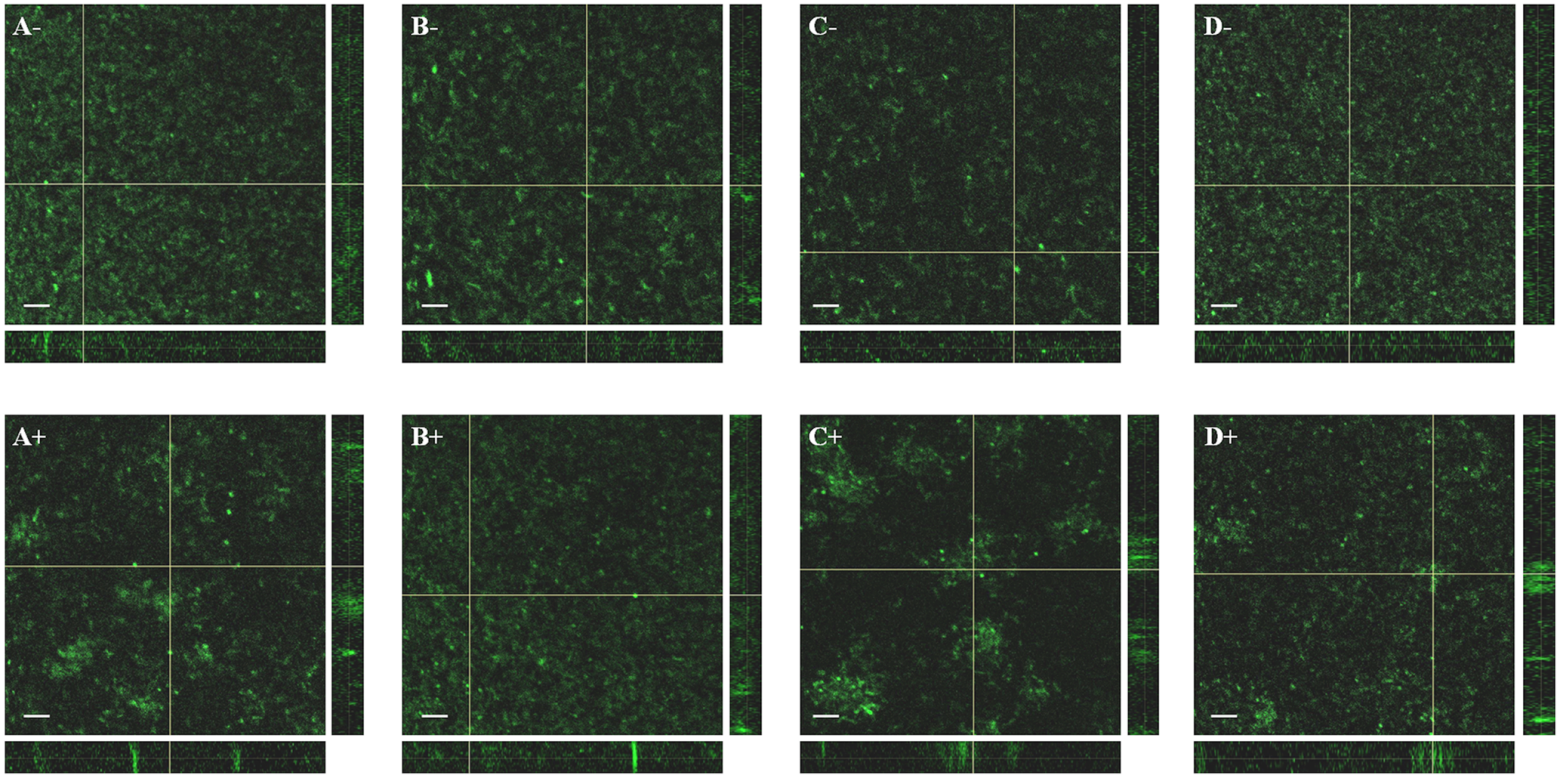
Biofilm structure of *S. fredii* SMH12 and derivatives on glass surfaces: reconstruction of the XY-axis, XZ-axis and YZ-axis. The top corresponds to cultures without flavonoids. The bottom corresponds to cultures with inducing flavonoid. A. SMH12. B. SVQ648. C. SMH12 (pME6863). D. SMH12 (pME6000). Bar, 20 µm. SMH12: wild-type, SVQ648: *nodD1* mutant, SMH12 (pME6863): lactonase strain. SMH12 (pME6000): carrying the empty plasmid.

When rhizobia colonize the legume root surface microcolonies or biofilms are formed. Could the observed colonization differences be a consequence of abnormal biofilm formation in the lactonase strain and in the *nodD1* mutant? To study the role of the biofilm formation during symbiosis, the biofilm structures both in the presence/absence of nod-gen inducing flavonoid (genistein) were observed using confocal microscopy experiments. Biofilm images of the wild-type, the *nodD1* mutant, the lactonase and the control strain carrying the empty plasmid, all labelled with GFP, were obtained (Fig. 2 and Fig. 3). Results showed that after 4 days of growth without flavonoid, the formed biofilm consisted of a monolayer in all the studied strains being the surface coverage statistically lower (about 50%) in the lactonase strain biofilm (Fig. 2c). In the presence of genistein, the formed biofilm changed from monolayer to microcolony type in SMH12, being the coverage statistically lower (almost 45%) (Fig. 2a). In the case of the *nodD1* mutant, no change was observed in the biofilm three-dimensional structure or surface coverage with genistein (Fig. 2b). Moreover, the lactonase strain showed a less surface-coverage (43%) without genistein with respect to the wild-type, but interestingly, in the presence of genistein the biofilm development underwent the transition to microcolony but no reduction in the coverage was obtained with respect to the lactonase strain without flavonoid (Fig. 2c). The control strain carrying the empty plasmid showed the same phenotype as the wild-type strain (Fig. 2d). These results suggest that, in *S. fredii* SMH12, the nodulation gene inducing flavonoids via NodD1 protein provoke a decrease in the bacterial attachment to an abiotic surface, since the monolayer biofilm structure (covering the whole surface) developed in the absence of flavonoid changed to microcolony-type (covering only some parts of the surface). Furthermore, these data indicate that QS signals must to be involved at least in the full formation of the monolayer-type biofilm on the glass surface.

### Influence of inducing flavonoids on the QS systems

Our earlier results demonstrated that *nod* gene inducing flavonoids increase the overall production of QS signals in *S. fredii* SMH12 [5]. To investigate if the activation of the QS systems takes place during the biofilm formation a quantification of the overall AI production was carried out in each condition and strain during the biofilm formation experiments on microtiter plates using the *A. tumefaciens* NT1 (pZRL4) biosensor. Firstly, the optimal concentration of bacterial supernatant required to obtain the wider differences between each condition without reaching saturation were determined adding different concentrations of bacterial supernatants (1%, 0.1% and 0.01%). The optimal concentration for these assays was 1% of the total volume of the biosensor culture (data not shown). Under these experimental conditions, results showed that the presence of genistein provoked a statistically significant higher production of AIs only in SMH12 (17% more), indicating that *nod* gene inducing flavonoids enhanced the QS signals accumulation via NodD1. Furthermore, β-galactosidase activity only in the presence of these flavonoids was similar to that obtained in the negative control (Table 4). As expected, a strong decrease in AI accumulation was detected in the supernatants from the lactonase strain, with or without genistein (Table 4). A supplementary TLC with extracts of the supernatants of biofilm experiments in microtiter plates showed a complete degradation of all AHLs in the lactonase strain. In the SMH12 strain carrying the empty plasmid no changes in the AHL profile were observed with respect to the wild-type strain (data not shown).

**Table 4:** β-galactosidase activity obtained using an adapted assay with *A. tumefaciens* NT1 (pZLR4) as bioreporter and grown in the presence of supernatants from biofilm cultures (1% v/v).

	Miller units	n (%)
Control (YM)	143.5±6.4	
Genistein (37 nM)	122.1±25.6	
Umbelliferone (62 nM)	131.2±7.2	
3-oxo-C6-HSL (5.5 µM)	824.7±21.2	
SMH12	717.6±23.6	100
SMH12+genistein (3.7 µM)	843.9±24.6	117*
SVQ648	702.7±28.4	97
SVQ648+genistein (3.7 µM)	745.8±39.2	104
SMH12 (pME6863)	150.2±53.8	21*
SMH12 (pME6863)+genistein (3.7 µM)	152.2±9.71	21*

Data are the mean ± SD of three independent experiments performed in triplicate.

n: percentage of induction of each supernatant with respect to SMH12 without flavonoids, defined as 100%.

Each β-galactosidase activity using biofilm supernatant was individually compared to that obtained in SMH12 without flavonoids by using the Mann-Whitney non-parametrical test. Numbers on the percentage of induction column followed by * are significantly different at the level α = 5%.

**Figure 4:**
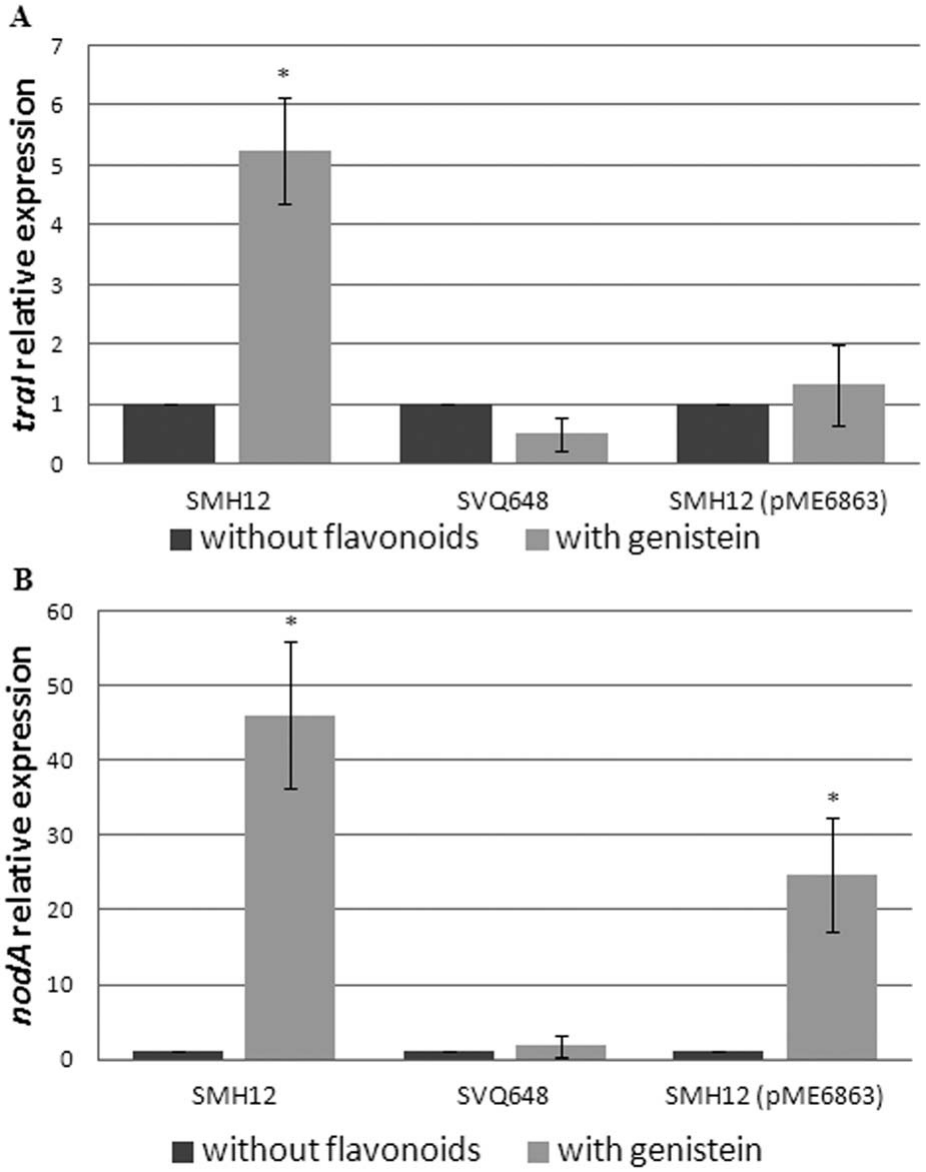
Quantitative RT-PCR analysis of the expression of *traI* and *nodA* from *S. fredii* SMH12 and derivatives from biofilm cultures. Expression data shown are the mean (± standard deviation of the mean) for three biological replicates. Expression was calculated relative to the expression without flavonoids of the wild-type strain by using the Mann-Whitney non-parametrical test. The asterisks indicate a significant different at the level α = 5%. White bars: biofilm cultures without flavonoids. Gray bars: biofilm cultures with genistein. A. *traI* relative expression. B. *nodA* relative expression. SMH12: wild-type, SVQ648: *nodD1* mutant, SMH12 (pME6863): lactonase strain.

Finally, to ascertain if the increase in AHL production with flavonoids in biofilm culture assays was correlated with an increase of gene transcription, a quantitative real time RT-PCR assays was carried out. Results showed that the expression of *traI,* an AHL-synthesis gene from SMH12 [5], significantly increased (5-fold) in the presence of genistein compared to the control without flavonoids. No changes in the *traI* expression were observed in the presence of flavonoid in the case of the *nodD1* mutant or in the lactonase strain, suggesting that the induction of the transcription takes place via NodD1 and that QS systems are necessary for the gene expression enhancement probably due to the typical positive feedback of the QS systems at high cellular density (Fig. 4a). As control, the expression of *nodA*, a gene positively regulated via NodD1 and flavonoids, was measured. As expected, a statistically significant increase was observed in *nodA* gene expression (46-fold and 24-fold, respectively) in both SMH12 and lactonase strains in the presence of genistein, but not in the *nodD1* mutant. Interestingly, the *nodA* gene expression in the lactonase strain in the presence of genistein was significantly lower than in the wild-type (Fig. 4b), which could explain both the reduced soybean nodulation and the less rizosphere attachment capacities of the lactonase strain, connecting the Nod factor production with these capacities (Table 2, Table 3, Fig. 1).

All these observations suggest that SMH12 *nod* gene inducing flavonoids enhance the transcription of certain AHL synthesis genes and the overall AHL production in biofilm experiments via NodD1 when the bacteria have reached the necessary cell density threshold.

### Role of the QS signals on biofilm formation

**Figure 5:**
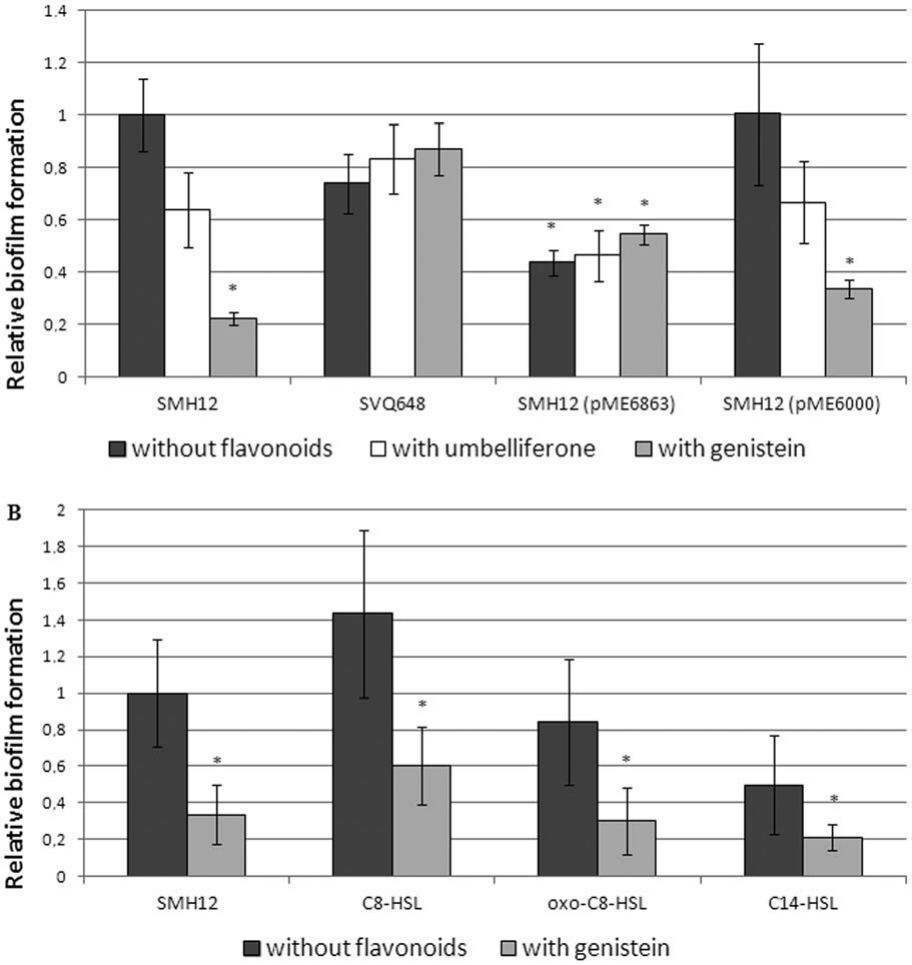
Adhesion of *S. fredii* SMH12 and derivatives on polystyrene surfaces. Biofilms were measured as the amount of crystal violet absorbed by the biofilm formed on multi-well plates and determined by absorbance at 570 nm after de-staining with ethanol (see methods). Absorbance of the wild-type strain was arbitrarily given a value of 1. Averages and standard deviations of eight replicas per strain corresponding to five independent experiments are shown. The asterisks indicate a significant different at the level α = 5% with respect to wild-type strain by using the Mann-Whitney non-parametrical test. A. Dark gray bars correspond to experiments performed without flavonoids, white to experiments with umbelliferone and light grey bars to experiments with genistein. B. Dark gray bars correspond to experiments performed without flavonoids and light grey bars to experiments with genistein. 3-oxo-C8-HSL and C8-HSL are used at 5.5 µM. C14-HSL is used at 55 µM. SMH12: wild-type, SVQ648: *nodD1* mutant, SMH12 (pME6863): lactonase strain. SMH12 (pME6000): carrying the empty plasmid.

Finally, for a more exhaustive analysis of the role of each QS signal in the biofilm formation, bacterial adhesion to polystyrene surface (biofilm formation experiments) was studied after 7 days of growing in low-phosphate MGM medium (Fig. 5). Firstly, it was confirmed that the glass coverage results obtained in confocal microscopy experiments are correlated with the bacterial adhesion values obtained in biofilm formation in polystyrene microtiter plate experiments. Thus, we could use this experimental approach to study the different biofilm structures. As shown in Figure 5, in the presence of genistein, SMH12 showed a significant reduction in the adhesion value to the abiotic surface (80%, where * is the statistical significance of the differences observed using the Mann–Whitney non-parametric test). Only a slight reduction was obtained using umbelliferone, a non-inducing flavonoid [5]. In the case of *nodD1* mutant, the adhesion values with or without flavonoids were similar to those obtained in the case of SMH12 without flavonoid. However, in the lactonase strain a lower adhesion was observed to polystyrene surfaces (50%) in all cases with respect to the wild-type strain without flavonoids, indicating that QS signals must be implied in the full differentiation of both types of biofilm on polystyrene surface since neither the high values without flavonoids nor the low values with inducing flavonoids are reached. To confirm that the differences observed in this strain were not due to the presence of the plasmid, the adhesion values were studied using SMH12 carrying the empty plasmid (pME6000). The experiment confirmed that this strain behaved like the wild-type strain (Fig. 5a). The absorbance at 600 nm after bacterial growth was similar in all strains and conditions (data not shown). Thus, the adhesion values to polystyrene surface in the wild type strain have a correlation with the values of surface coverage obtained on a glass surface with confocal microscopy experiments (Fig. 2).

Once this method was validated as a reporter of the formation of the different biofilm types (monolayer with high values of coverage/adhesion and microcolony with low values of coverage/adhesion), the influence of QS signals in the biofilm formation of SMH12 with or without inducing flavonoids was studied. For this purpose, the cognate AHLs of SMH12, 3-oxo-C8-HSL, C8-HSL and C14-HSL [5], were added to wild-type bacterial cultures at physiological concentrations (data not shown) in biofilm formation experiments on microtiter plates (Fig. 5b). A slight increase or decrease in the adhesion to the polystyrene surface was observed when the medium was supplemented with C8-HSL or C14-HSL, respectively. However, these differences were not statistically significant (Fig. 5b). The absorbance at 600 nm after bacterial growth was similar in all bacteria and conditions (data not shown).

## Discussion

During the first stages of root colonization, leguminous plants exudate molecules which chemotactically attract rhizobia. Once in the rhizosphere, the rhizobial population colonizes the root surface, forming a bacterial community surrounded by a matrix produced by its own bacteria, the biofilm. In the family *Rhizobiaceae*, the bacterial intrinsic factors that are required for the biofilm formation are mainly EPS and bacterial QS systems as well as in the case of *S. meliloti* the Nod factor production [12]. A fundamental question is whether the process of biofilm formation significantly affects nodulation in the symbiosis *S. fredii* SMH12-*G. max* cv. Osumi. To answer this question, the significance of QS systems and the *nod* gene inducing flavonoids on the biofilm formation, the colonization and the symbiosis with soybean were studied by means of both a *nodD1* mutant and a lactonase strain of *S. fredii* SMH12.

As expected, nodulation tests with soybean showed that the symbiotic phenotype of the *nodD1* mutant is similar to that obtained in non-inoculated plants, since this bacterium is unable to detect via NodD1 the flavonoids exuded by plants. Consequently the Nod factor production is blocked. Interestingly, results indicate that SMH12 QS systems could regulate some important process during the symbiosis, since the lactonase strain showed reductions in both the plant top dry mass and the fresh mass of nodules formed with respect to the wild-type strain but only in the number of nodules the reduction was statistically significant (Table 2). As described in the introduction, QS regulates a broad variety of phenotypes, including the biofilm formation. In the review of Rinaudi and Giordano [12] the mechanisms involved in both the rhizobial biofilm formation and the attachment to the plant roots are summarized. Taking into consideration the previous reports, they concluded that the biofilm lifestyle allows rhizobia to survive under unfavourable conditions and in certain species the biofilm formation is clearly an important feature of symbiotic ability [19], [22], [23], [24]. However, the role and regulation of biofilm formation during the symbiosis *S. fredii* SMH12-soybean has not been reported. Our results suggest that there is a correlation between QS systems, biofilm formation, root colonization and symbiosis. Firstly, three independent experiments of bacterial root colonization showed a significant reduction in bacterial root colonization in the *nodD1* mutant and especially in the lactonase strain. These differences were more visible in the lateral root colonization (Table 3). Interestingly, in addition to the differences in the bacterial number, these two strains showed more of a spread surface distribution on roots compared to the wild-type, in which the bacteria appeared clustered (Fig. 1). These observations would indicate that the *nodD1* mutant and specially the lactonase strain do not colonize the root surface optimally due to an alteration in their biofilm formation ability. The impaired biofilm formation in the case of the lactonase strain would explain its less nodulation capacity in the soybean test (Table 2).

To corroborate this hypothesis, the biofilm structure was analyzed by confocal microscopy. Two types of biofilms were observed, the monolayer-type in the absence of flavonoid (bacterial population cover the entire surface homogeneously), and the microcolony-type in the presence of genistein (bacterial population is clustered) (Fig. 2a and Fig. 3a). These observations would indicate that on the surface of the legume root, with a high concentration of flavonoids, the bacterial biofilm could undergo a transition from monolayer to microcolony. Perhaps this biofilm structure allows the bacterial colonization of specific root areas (those with high flavonoid exudation), which would be the optimal for the symbiosis initiation (i.e. root hairs).

Furthermore, analysing these results we infer that QS systems is involved at least in the formation of the monolayer type, since the lactonase strain developed incomplete biofilm phenotypes without inducing flavonoids (Fig. 2c and Fig. 3c). In fact, most rhizobial biofilms investigated so far are regulated directly or indirectly by QS systems [12]. Interestingly, the transition to microcolony-type biofilm requires Nod factor production since this biofilm was not developed in the presence of genistein in the *nodD1* mutant (Fig. 2b and Fig. 3b). Fujishige *et al.* [19] reported that *nodD1ABC* of *S. meliloti*, involved in the synthesis of Nod factors, are necessary for the three-dimensional architecture of the biofilm and, in fact, these molecules are present in the biofilm matrix. The mutation of any of these genes generates a monolayer-type biofilm. However, in contrast with our results, the presence of inducing flavonoids is not necessary for the development of three dimensional structures in the biofilm of *S. meliloti* [19].

Interestingly, our findings also suggest that flavonoids, beyond inducing the synthesis of Nod factors (which integrate the symbiotic biofilm matrix), are enhancing the QS systems in biofilm formation experiments (Table 4 and Fig. 4a). In the presence of inducing flavonoids, an increase in the *traI* expression and in the overall AI production was detected. These effects were not observed in the lactonase strain due to the positive feed-back regulation of the QS systems which only occurs when the threshold in cellular density is reached [6] (Fig. 4a and Table 4). Furthermore, it is clear that the enhancement of the *traI* gene expression takes place via NodD1, because in the *nodD1* mutant this over expression was not observed. He et al. [38] studied the QS systems of *Rhizobium* sp. NGR234, a *Sinorhizobium fredii* related bacterium, but they only unequivocally identified the *tra* system. This QS system is homologous to the one responsible for the plasmid Ti transfer in *A. tumefaciens.* This transference occurs only in the presence of opines, compounds that are produced by plants. Control of the Ti plasmid transference is modulated by TraM, a small protein that binds to and inactivates TraR, which senses the AI concentration. The induction in the presence of opines allows the synthesis of TraR at levels that overcome the inhibitory activity of TraM, activating the QS systems and the plasmid transfer [39]. In NGR234, the over expression of TraR activates not only the *tra* system but also other QS systems of the bacteria, since an increase in the overall AI production was detected [34]. However, so far, no natural compounds exuded by plants (similar to opines for *A. tumefaciens* QS systems) have been identified as inductor molecules for NGR234. Interestingly, results obtained in this paper and those reported in our previous work [5] indicate that the *nod* gene inducing flavonoids could be these inducing compounds for *S. fredii* SMH12.

In summary, experiments show that SMH12 QS systems must be involved at least in the full differentiation of monolayer-type biofilm and, moreover, in the presence of inducing flavonoids, these systems increase the AHL production and the biofilm undergoes a transition to microcolony-type (Fig. 4a, Table 4 and Fig. 2). Logically, this expression increase in the presence of flavonoids could be related to the developement of the microcolony-type biofilm, which would only occur in the rhizosphere. This fact would allow, through a unique key molecule, the efficient colonization of the legume root and the activation of the synthesis of Nod factors, both required for successful symbiosis. Interestingly, the study of the role of QS signals on biofilm formation experiments (Fig. 5) showed no statistically differences in adhesion values with or without flavonoid after addition of the wild-type cognate AHLs (Fig. 5b). However, in the case of the lactonase strain, the decrease in the adhesion values in the presence of inducing flavonoids did not reach the values obtained in the wild-type strain. This result suggests that, despite the transition to microcolony-type biofilm is regulated directly by NodD1 protein and inducing flavonoids (Fig. 2), the QS signals could also be involved in the full differentiation of this biofilm on the polystyrene surface (Fig. 5a).

**Figure 6:**
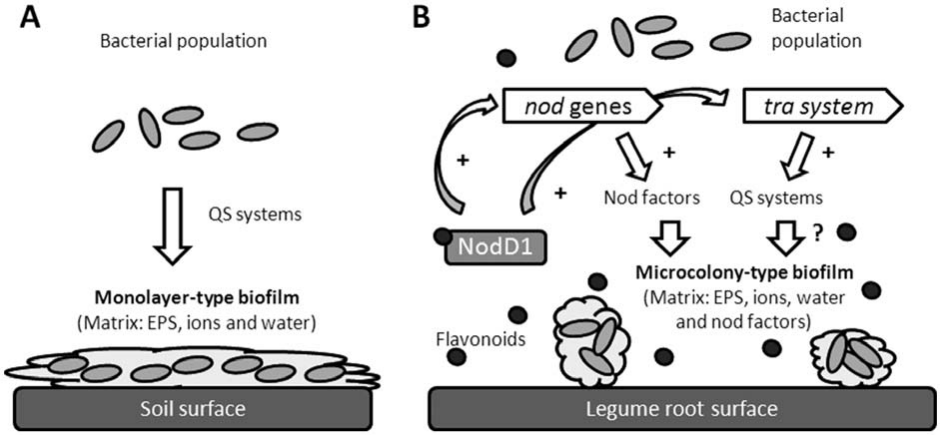
Model of biofilm formation in *S. fredii* SMH12. A. In soil. B. In rizosphere. *S. fredii* SMH12 forms monolayer-type biofilm when colonize soil surfaces. When the bacterium colonizes the legume root (in presence of inducing flavonoids), it forms microcolony-type biofilm, which is necessary for a successful root colonization and symbiosis between rhizobia and legume.

Thus, taking into consideration all the results, we propose the following model (Fig. 6): *S. fredii* SMH12 forms a QS-regulated monolayer-type biofilm (whose matrix would be mainly composed of EPS, water and ions) when bacterium colonizes soil. In the rhizosphere, the exudation of inducing flavonoids, besides inducing the synthesis of Nod factors which are necessary for the microcolony-type biofilm, potentiate via NodD1 the *tra* QS system, which leads to an overproduction of the QS signals. This accumulation and the synthesis of Nod factors are required for the full development of the microcolony-type biofilm, whose matrix should be composed of EPS, water, ions and Nod factors. The complete formation of this symbiotic biofilm is necessary for a successful root colonization and symbiosis between rhizobia and legume.

Despite demonstrating in this work that the rhizobial biofilm formation is important for colonization and symbiosis of *S. fredii* SMH12 with the soybean, there are still many aspects that must to be studied in the future to further clarify this process.
